# Post-operative myocardial infarction following aortic root surgery with coronary reimplantation: a case series treated with percutaneous coronary intervention

**DOI:** 10.1093/ehjcr/ytz181

**Published:** 2019-10-22

**Authors:** Carly Adamson, Paul Rocchiccioli, Richard Brogan, Colin Berry, Thomas J Ford

**Affiliations:** 1 West of Scotland Heart and Lung Centre, Golden Jubilee National Hospital, Clydebank, G81 4DY, UK; 2 British Heart Foundation, Glasgow Cardiovascular Research Centre, Institute of Cardiovascular and Medical Sciences, University of Glasgow, Glasgow, G12 8TA, UK; 3 Department of Cardiology, Gosford Hospital, NSW, Australia; 4 University of New South Wales, Sydney, Australia

**Keywords:** Aortic root replacement, Bentall procedure, David procedure, Percutaneous coronary intervention, Coronary reimplantation, Myocardial infarction, Case series

## Abstract

**Background:**

Coronary ostial stenosis is an uncommon but potentially lethal complication following aortic root replacement with or without aortic valve replacement (including Bentall and David procedures). This manifests clinically as acute myocardial ischaemia in the early or late post-operative period. Traditionally, this might be managed with redo open-heart surgery.

**Case summary:**

This case series describes two presentations where urgent percutaneous coronary intervention was used to manage myocardial infarction complicating aortic root surgery with coronary reimplantation.

**Discussion:**

This series highlights the risk of acute myocardial infarction after cardiac surgery involving coronary reimplantation. Emergency percutaneous coronary intervention is feasible and illustrates the importance of shared post-operative care involving the cardiac surgeons and the cardiology team.

**Learning points**

- Acute myocardial infarction is an unusual complication in the days to months following aortic root replacement with coronary reimplantation (including Bentall and David procedures) and can be due to obstruction of the reimplanted artery. PCI can be considered as a salvage treatment for these patients.
- Cardiothoracic centres should perform complex aortic root surgery within the setting of an experienced multidisciplinary team involving cardiac surgeons, cardiologists with allied health professional input.

## Introduction

Coronary ostial stenosis is an uncommon but recognized complication of aortic root surgery involving reimplantation of the coronaries to a graft and usually presents within the first 6 months following aortic root surgery.^1^ Ischaemia may become apparent immediately post-procedure, and this is generally managed with immediate reoperation with resiting of coronary button or bypass grafting.^2^ However, immediate grafting may not always be possible for technical reasons and decisions about revascularization become more complicated in patients who develop acute ischaemia in the weeks following the index procedure. We report a case series where urgent percutaneous coronary intervention (PCI) was used in the setting of acute myocardial infarction in patients who had recently undergone aortic root replacement as a valve-sparing procedure. The purpose of this report is to raise awareness of this complication and utility of PCI as a treatment option. This is particularly important for patients where surgery may not be technically possible or those who present in extremis.

## Timeline

**Table:** 

	Case 1	Case 2
Indication for operation	Marfan’s syndrome. Dilated aortic root at 5.7 cm	Bicuspid aortic valve with aortic regurgitation (AR). Worsening symptoms with progressive AR and dilated aortic root at 7 cm
Time from diagnosis to operation	2 months	3 weeks
Operation	Valve sparing root replacement (David procedure)	Aortic root replacement and repair of native valve
Immediate post- operative recovery	Unremarkable, discharged 5 days later	Angina type pain on mobilizing
Time: index operation to ischaemic presentation	2 weeks	4 days
Diagnosis of coronary ostial stenosis	Inferior ST-elevation myocardial infarction. IVUS confirmed extrinsic compression of neo-ostium.	Computed tomography coronary angiography suggests mechanical distortion of proximal right coronary artery (RCA)
Procedure	1 × drug-eluting stent (DES) to proximal RCA	1 × DES proximal RCA
Follow-up	Echocardiography (ECHO) shows moderate left ventricular (LV) systolic dysfunction, with fatigue being only ongoing symptom	No ongoing symptoms, most recent ECHO shows mild left ventricular systolic dysfunction

## Case presentation 

### Patient 1

This 38-year-old woman with background of Marfan’s syndrome was admitted to hospital following an episode of syncope. Echocardiography (ECHO) showed severe dilation of the ascending aorta with mild mitral and aortic regurgitation (AR) and normal left ventricular systolic function. Computed tomography confirmed dilation at 57 mm. Valve sparing aortic root replacement (David procedure) was carried out 2 months later after which she made an unremarkable recovery, being discharged home 5 days post -operatively. She was not on any regular medications prior to admission and refused any medications on discharge from hospital.

**Figure 1:**
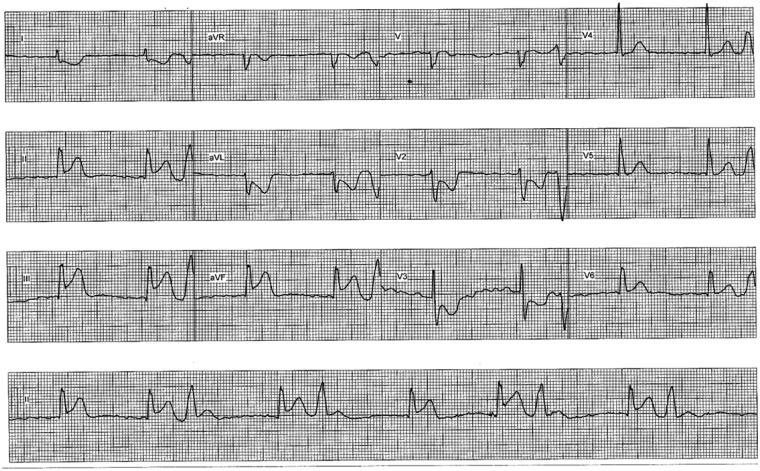
Electrocardiogram taken on admission to catheterization lab after patient admitted directly from home through the optimal reperfusion service. Electrocardiogram shows an inferior-posterior ST-elevation myocardial infarction.

**Figure 2:**
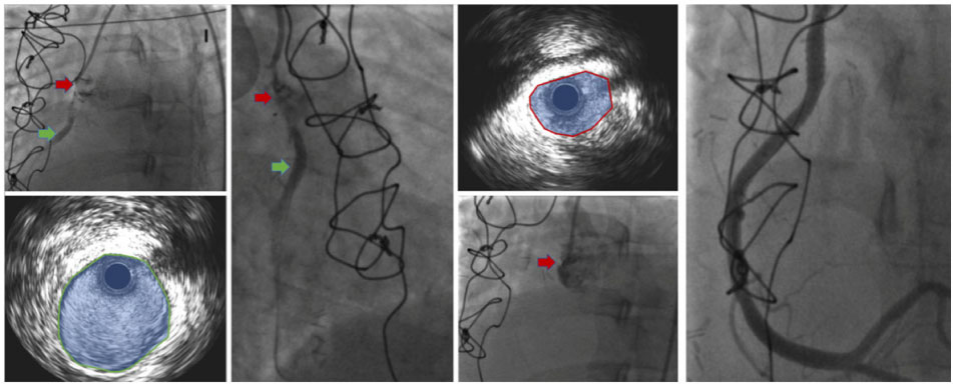
A 38-year-old woman with Marfan’s syndrome underwent elective David procedure. Ten days post-operatively, she developed an inferior ST-segment elevation myocardial infarction. The emergency coronary angiography shows extrinsic compression of the neo-ostium with resultant thrombosis of the right coronary artery. The right sided projection of the coronary artery shows the compression and thrombus (red arrow) corresponding with IVUS image showing thrombus and reduced luminal area. The mid-right coronary artery is large and normal without any atherosclerosis or compression (green arrow with corresponding IVUS image, bottom left). A drug-eluting stent implantation was successful with excellent final angiographic result.

She was readmitted 10 days post -operatively directly from home to interventional cardiology via our regional optimal reperfusion service with an inferior ST-elevation myocardial infarction (STEMI) and cardiogenic shock [electrocardiogram (ECG) shown in *Figure 1*]. Prior to this, she had experienced a short history of exertional chest pain. Angiography showed the right coronary artery (RCA) was occluded proximally with thrombus and appearance of mechanical disruption of the aorto-ostial anastomosis (*Figure 2*). Reperfusion was achieved after wiring and export of a large thrombus, followed by ventricular fibrillation (VF) cardiac arrest after which an intra-aortic balloon pump was inserted and the patient intubated. After discussion with the multidisciplinary team (MDT) in the catheterization lab, the decision was to proceed with intravascular ultrasound (IVUS)-guided percutaneous intervention. IVUS confirmed extrinsic mechanical compression at the right coronary neo-ostium with no atherosclerosis present. This was successfully treated with one drug-eluting stent (DES) (*Figure 2*). Emergency ECHO in the catheterization lab showed severe right and left ventricular systolic dysfunction (LVSD). She was extubated the next day. Echocardiography on the 8th day of admission showed a dilated left ventricle with moderate segmental function and notably dilated failing right ventricle. Her recovery was complicated by ventilator acquired pneumonia and atrial fibrillation (AF) and she was discharged home after 3-week hospital admission.

At 18 months of follow-up, her symptoms and ventricular function had significantly improved [New York Heart Association I–II and mild systolic impairment without right ventricular (RV) impairment].

### Patient 2

This 53-year-old gentleman with known bicuspid aortic valve and dilated aortic root developed decompensated heart failure related to severe AR in the setting of severely dilated aortic root (70 mm). His left ventricular (LV) function was moderately impaired which improved with medical therapy prior to operation. Computed tomography coronary angiography (CTCA) showed trivial plaque without obstructive coronary artery disease. Three weeks later after optimizing his medical treatment, he underwent aortic valve repair with valve-sparing aortic root replacement (David procedure). Post-procedure ECHO showed no AR with good LV function. Aspirin was started in the post -operative period with plan to continue for 3 months. After developing AF, he commenced on treatment dose low- molecular-weight heparin, amiodarone, and beta-blocker. His other medications included an angiotensin-converting enzyme inhibitor, statin, and omeprazole.

**Figure 3:**
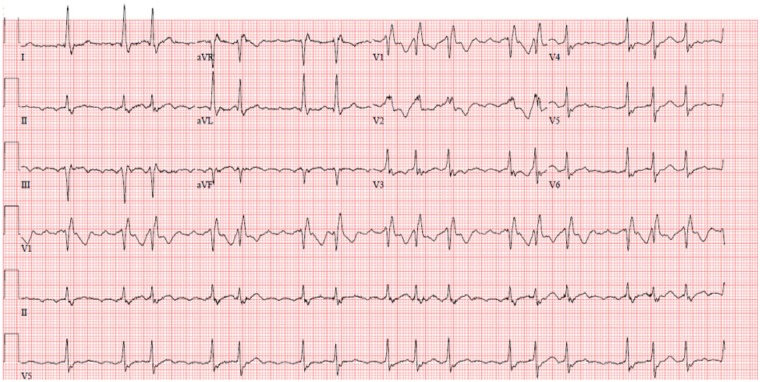
Electrocardiogram taken in recovery period from David procedure when the patient complained of chest pain while mobilizing. Electrocardiogram shows new right bundle branch block and new atrial flutter with uncontrolled ventricular rate.

**Figure 4:**
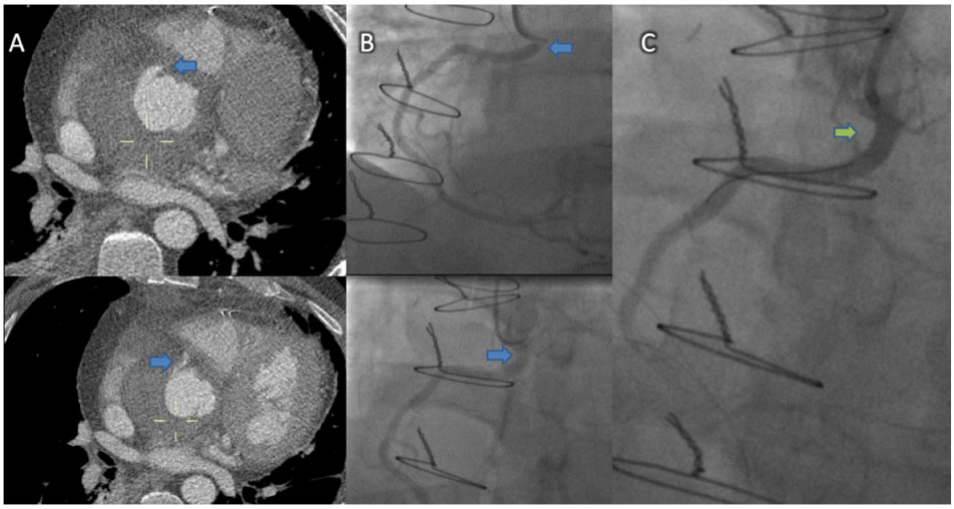
A 53-year-old man with a bicuspid aortic valve underwent elective cardiac surgery involving the David procedure. Four days post-operatively developed non-ST-elevation myocardial infarction. Gated computed tomography angiography suggested distortion of the proximal right coronary artery marked with a blue arrow (*A*). This corresponds with the stenosis marked with blue arrows in diagnostic invasive angiography images (*B*). There was a good result following implantation of a drug-eluting stent with the stented area marked by the green arrow (*C*).

His inpatient recovery was complicated by recurrent chest pain on mobilizing. Levels of high-sensitivity troponin remained elevated at levels higher than expected in the post-operative period ( high-sensitivity troponin T peak 489 ng/mL) raising clinical concern regarding ongoing ischaemia. ECG showed AF with an uncontrolled ventricular rate and new right bundle branch block (*Figure 3*). Therefore, a CTCA was performed which was suggestive of distortion of the proximal RCA (*Figure 4*). Urgent invasive angiography showed proximal RCA stenosis which was treated with one DES with good angiographic results. Echocardiography at Day 5 showed basal to mid-infero-lateral akinesia with overall moderate LVSD. The right ventricle size was normal with mildly impaired systolic function. Recovery was complicated by lower respiratory tract infection and ongoing AF but he was discharged home 12 days later. At clinic review 2 months post-operatively, he was well with no symptoms of angina or syncope and with mild shortness of breath only on moderate exertion. At follow-up, there was moderate segmental LVSD (consistent with the target vessel infarction), whilst RV function was normal.

## Discussion

Composite valve-graft replacement of the aortic root including variations on the Bentall procedure has traditionally been considered the gold standard surgical approach for treatment of aortic root dilatation. When it is possible to retain the native valve, valve-sparing procedures such as the David procedure may improve quality of life and avoid complications relating to degeneration of the bioprosthesis and issues with anticoagulation.^3^ Both approaches require reimplantation of the coronary buttons.

Coronary ostial stenosis is a rare but recognized complication of aortic root surgery involving reimplantation of the coronaries. Incidence is difficult to ascertain but has previously been given as between 5% and 6% in original Bentall procedure^1^ and between 1% and 5% for patients undergoing aortic valve replacement only.^4^ A systematic review of the Bentall procedure gave an early mortality of 5.6% with 5.9% of these caused by myocardial infarction,^5^ some of which may be caused by early coronary ostial obstruction.

There are several distinct mechanisms of ostial obstruction proposed. These include damage during instrumentation and delivery of cardioplegia and issues with sutures to the coronary button.^2^ Hardening and organization of clot could cause mechanical compression. Positioning of the anastomosis can result in tension through the coronary.^6^ Careful reimplantation of the coronary button is likely to reduce this risk.^7^ Specific techniques to facilitate tension-free button attachment include identifying the site for the RCA button after the anastomosis of the distal graft to the aorta is complete although there is debate about the best approach.^6^^,^^8^ Others have suggested potential undiagnosed coronary artery disease or development of oedema at the coronary reimplantation site.^9^ Surgical glue used to secure the anastomosis has also been implicated, either by directly exerting external pressure^10^ or through abnormal patient inflammatory response to the glue.^11^ Coronary vasospasm is another potential cause of ischaemia in patients following bypass surgery and is a common cause of angina.^12^

There are several individual case reports describing the use of PCI in patients who present with ischaemia having undergone aortic root replacement with coronary button re-implantation. This case series demonstrates two heterogeneous presentations linked by the choice of PCI as a salvage option for emergency myocardial revascularization. Each of these cases was discussed with the multidisciplinary team including cardiologists, cardiothoracic surgeons, and cardiac anaesthetists. The collective decision was that PCI was felt to be the most appropriate management in the emergency setting.

The mechanism of ischaemia related to dynamic coronary obstruction is likely to have occurred subacutely given that RCA occlusion and related RV infarction would prohibit weaning off bypass. The options of surgical re-intervention may have advantages and is potentially less challenging than late redo surgery due to lack of adhesions. A mechanical complication (e.g. excess tension through the ostium or external compression by haematoma or surgical glue) may be identified and corrected including possible refashioning of the coronary button. If this is not possible bypass may be an alternative, however, long-term outcome of a graft may be reduced given the potential for competitive flow through the coronary ostium which would vary through the cardiac cycle.

PCI was felt to offer the best emergency salvage and the acute success of the procedure was supported by prompt improvement in haemodynamics with ST-segment resolution. In general, minimization of time to revascularization is the primary driver behind choice of revascularization in STE MI with cardiac bypass surgery being considered when the patient is not suitable for PCI.^13^ In this situation, the dynamic change on the stent may lead to altered wall stress and propensity to restenosis or stent fracture in the longer term. Clearly, more evidence is needed with careful longer-term follow-up against repeat surgical button reimplantation as the gold standard approach. Cardiovascular teams looking after these patients will need to be vigilant to any symptoms suggestive of ischaemia.

Cardiogenic shock in these cases may result from the target vessel myocardial infarction with contribution from potential suboptimal myocardial protection which is of vital importance in lengthy aortic root reconstructions (e.g. the lengthy David procedure).^14^

Of note, Patient 1 was not on antiplatelet or anticoagulation at time of discharge postoperatively having refused medications. Patient 2 was started on Aspirin post -operatively with a plan to continue for 3 months. Often post -operative patients would be treated with either antiplatelet or anticoagulation for 3 months to reduce the risk of thromboembolic complication and this may have contributed to the acute presentation of Patient 1 .^15^

Alongside early revascularization, both patients were treated with IV fluid to expand plasma volumes and treat RV infarction. In patients with RV infarction, hypotension and absence of pulmonary oedema should be treated with plasma expansion, ideally with invasive monitoring. Both these patients also had a degree of LV impairment which can make resuscitation more challenging as RV dilatation can increase diastolic LV pressures and contribute to low output state.^16^ Case 1 demonstrated the use of intra-aortic balloon pump counter pulsation in initial management of RV infarction. Intra-aortic balloon pump can improve haemodynamics in this setting.^17^ Given the acuity of her presentation the ECMO team were activated in anticipation of the need to further escalate her care.

These cases demonstrate the potential efficacy of PCI in management of both emergency STEMI and a high-risk non-STEMI. Prompt diagnosis and early multidisciplinary team discussion including with the operating surgeon allowed the patients to receive early and successful revascularization with PCI. Good communication was key to each of these decisions specifically collaboration between the surgical and cardiology teams.

## Conclusions

Acute myocardial infarction is an uncommon but important complication following aortic root replacement with coronary reimplantation. Extrinsic compression or distortion of the replanted coronary neo-ostium may result in vessel occlusion. Urgent PCI may facilitate emergency revascularization in these patients.

## Lead author biography

**Figure:**
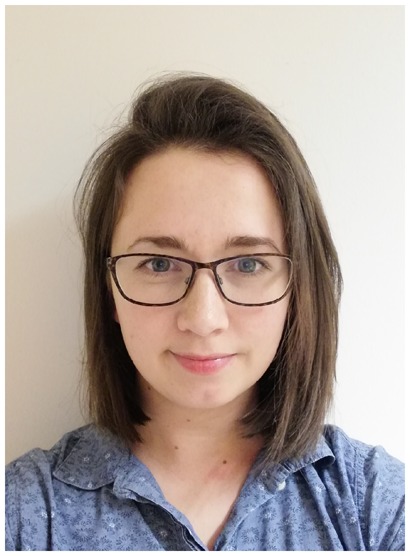


Dr Carly Adamson graduated with MBChB from the University of Dundee in 2013. She has developed a keen interest in pursuing a career in cardiology during general medical training and is currently working as a Cardiology Clinical Fellow.

## Supplementary material

Supplementary material is available at *European Heart Journal - Case Reports* online.

## Funding

This study received grant funding from the British Heart Foundation (RE/13/5/30177).

**Slide sets:** A fully edited slide set detailing this case and suitable for local presentation is available online as Supplementary data.

**Consent:** The author/s confirm that written consent for submission and publication of this case report including image(s) and associated text has been obtained from the patient in line with COPE guidance. 

**Conflict of interest:** none declared.

## Supplementary Material

ytz181_Supplementary_Slide_Set
