# Supplementary material for: Post-operative myocardial infarction following aortic root surgery with coronary reimplantation: a case series treated with percutaneous coronary intervention
Source: Eur Heart J Case Rep. 2019 Oct 22;3(4):1–6. doi: 10.1093/ehjcr/ytz181 (PMC7042144; doi:10.1093/ehjcr/ytz181)
Supplement: ytz181_Supplementary_Slide_Set [file ytz181_supplementary_slide_set.pptx]

## Slide 1
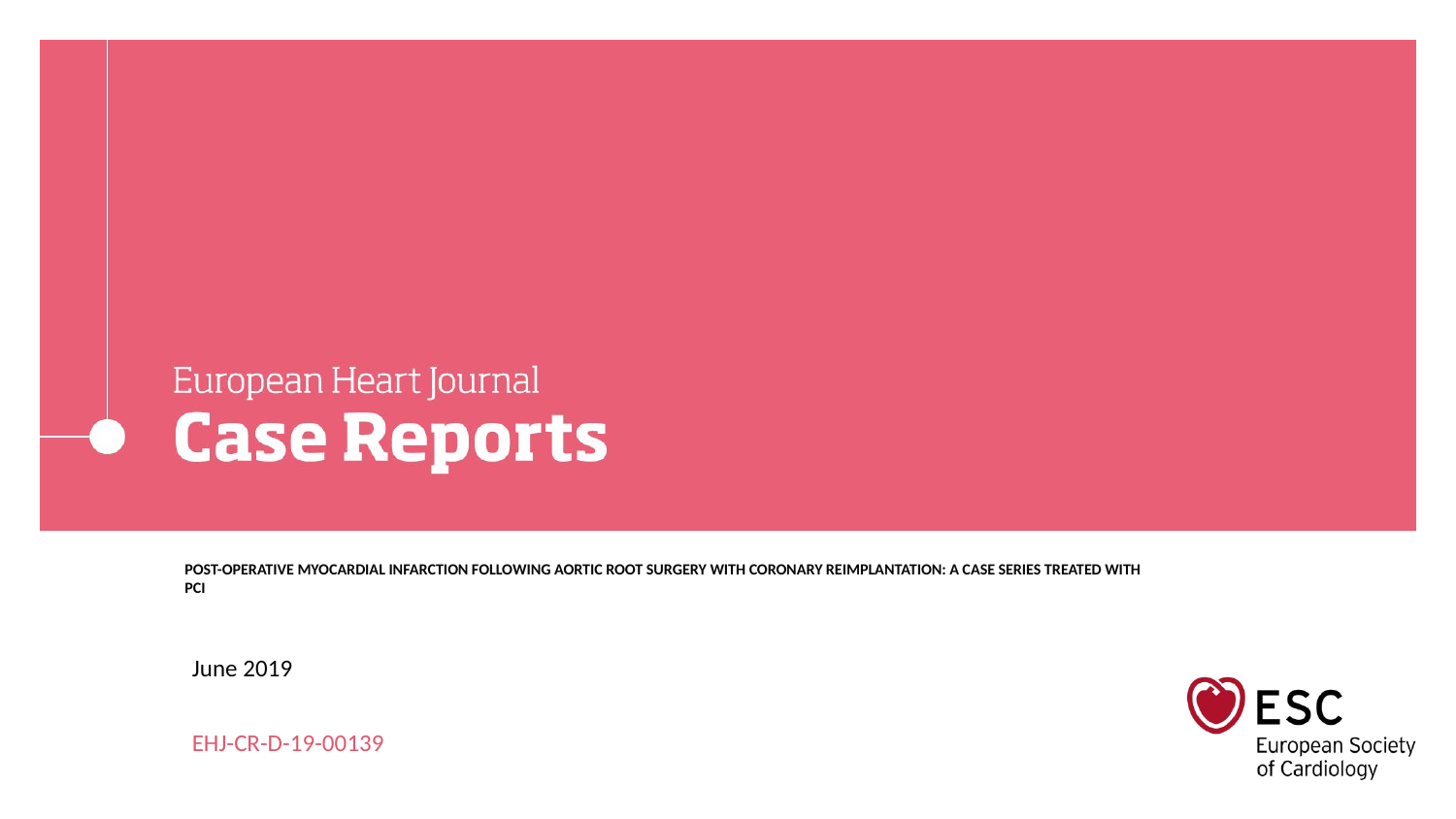

# Post-operative myocardial infarction following aortic root surgery with coronary reimplantation: a case series treated with PCI
June 2019
EHJ-CR-D-19-00139

## Slide 2
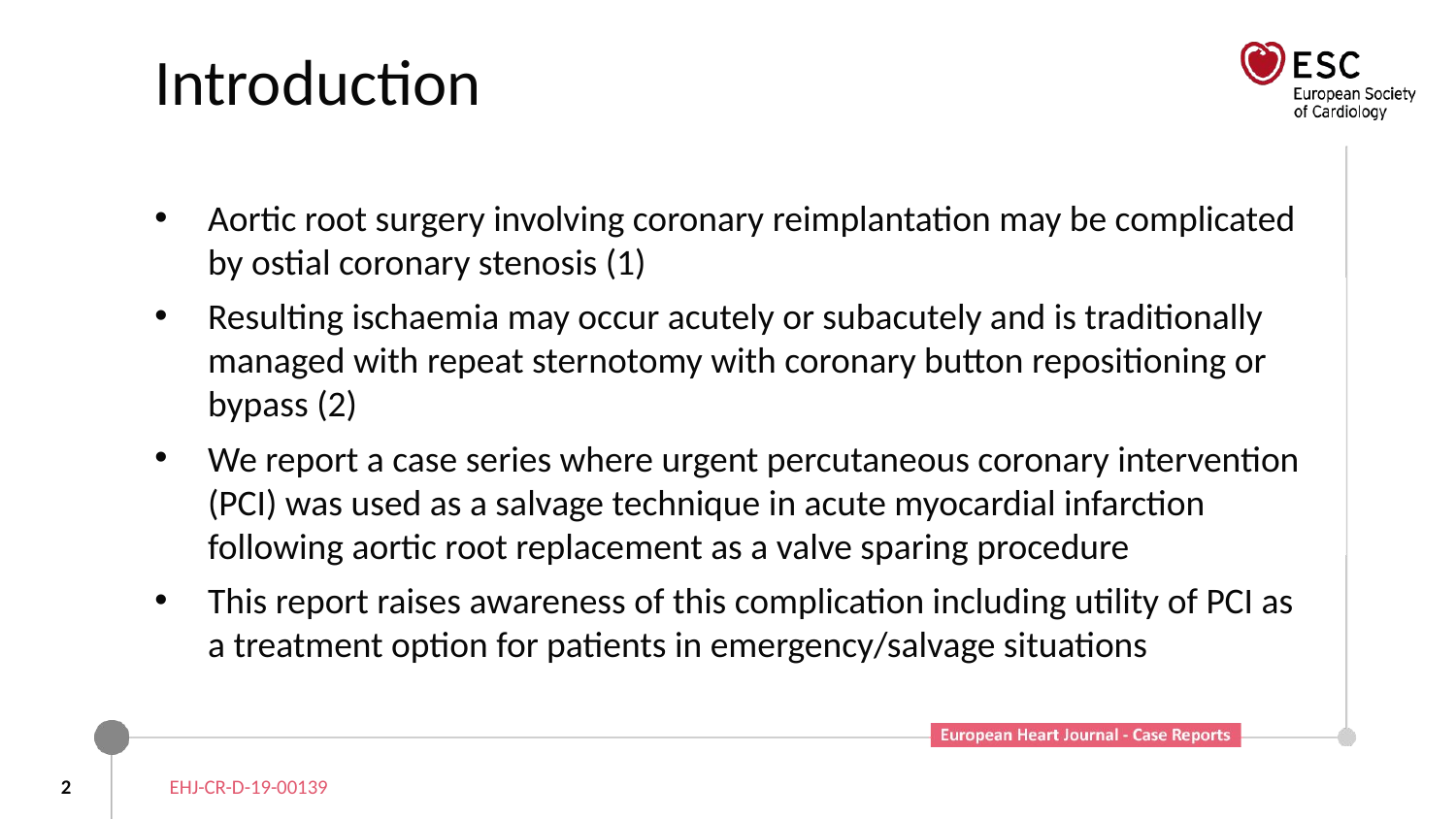

# Introduction
Aortic root surgery involving coronary reimplantation may be complicated by ostial coronary stenosis (1)
Resulting ischaemia may occur acutely or subacutely and is traditionally managed with repeat sternotomy with coronary button repositioning or bypass (2)
We report a case series where urgent percutaneous coronary intervention (PCI) was used as a salvage technique in acute myocardial infarction following aortic root replacement as a valve sparing procedure
This report raises awareness of this complication including utility of PCI as a treatment option for patients in emergency/salvage situations
2
EHJ-CR-D-19-00139

## Slide 3
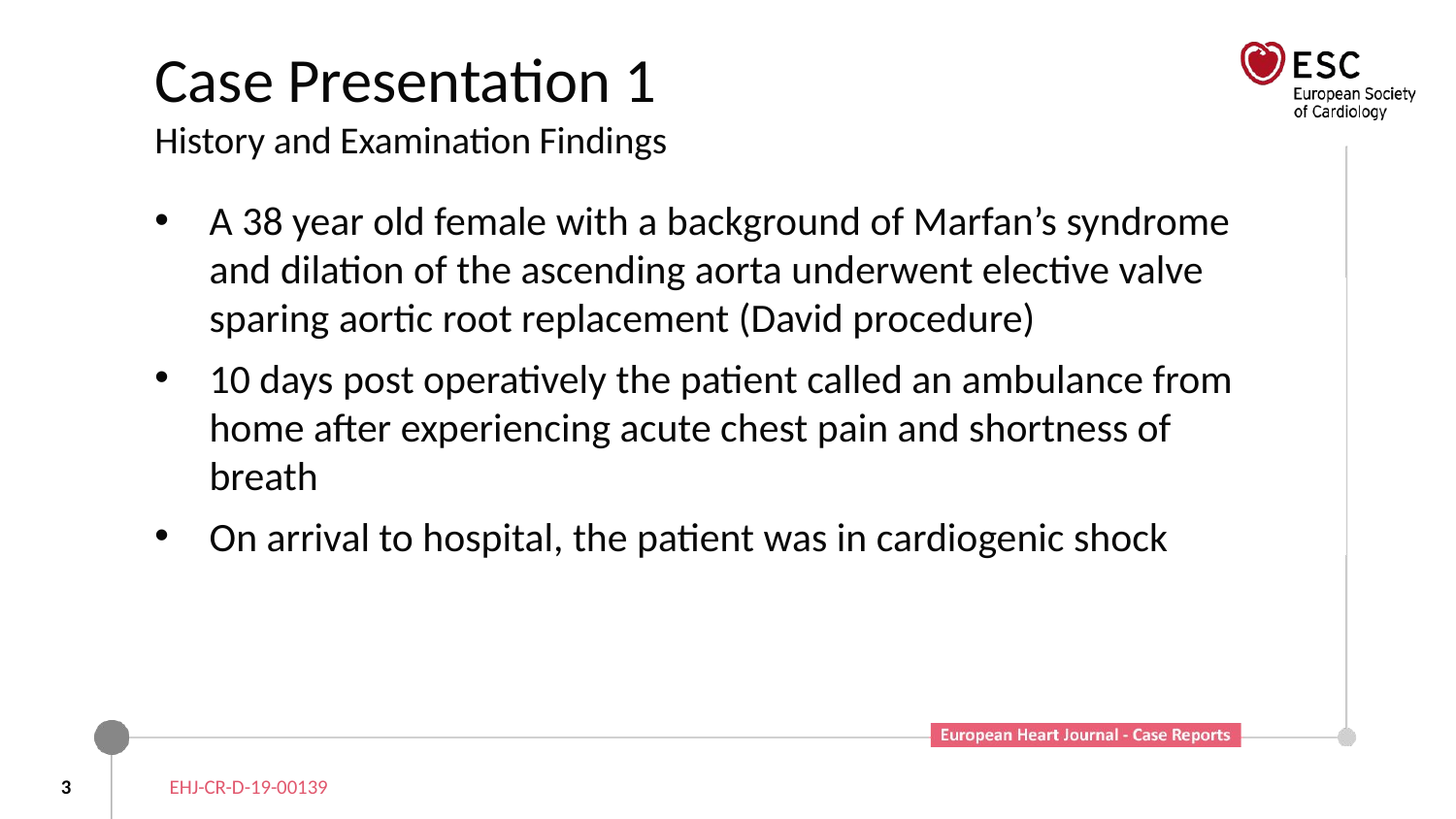

# Case Presentation 1 History and Examination Findings
A 38 year old female with a background of Marfan’s syndrome and dilation of the ascending aorta underwent elective valve sparing aortic root replacement (David procedure)
10 days post operatively the patient called an ambulance from home after experiencing acute chest pain and shortness of breath
On arrival to hospital, the patient was in cardiogenic shock
3
EHJ-CR-D-19-00139

## Slide 4
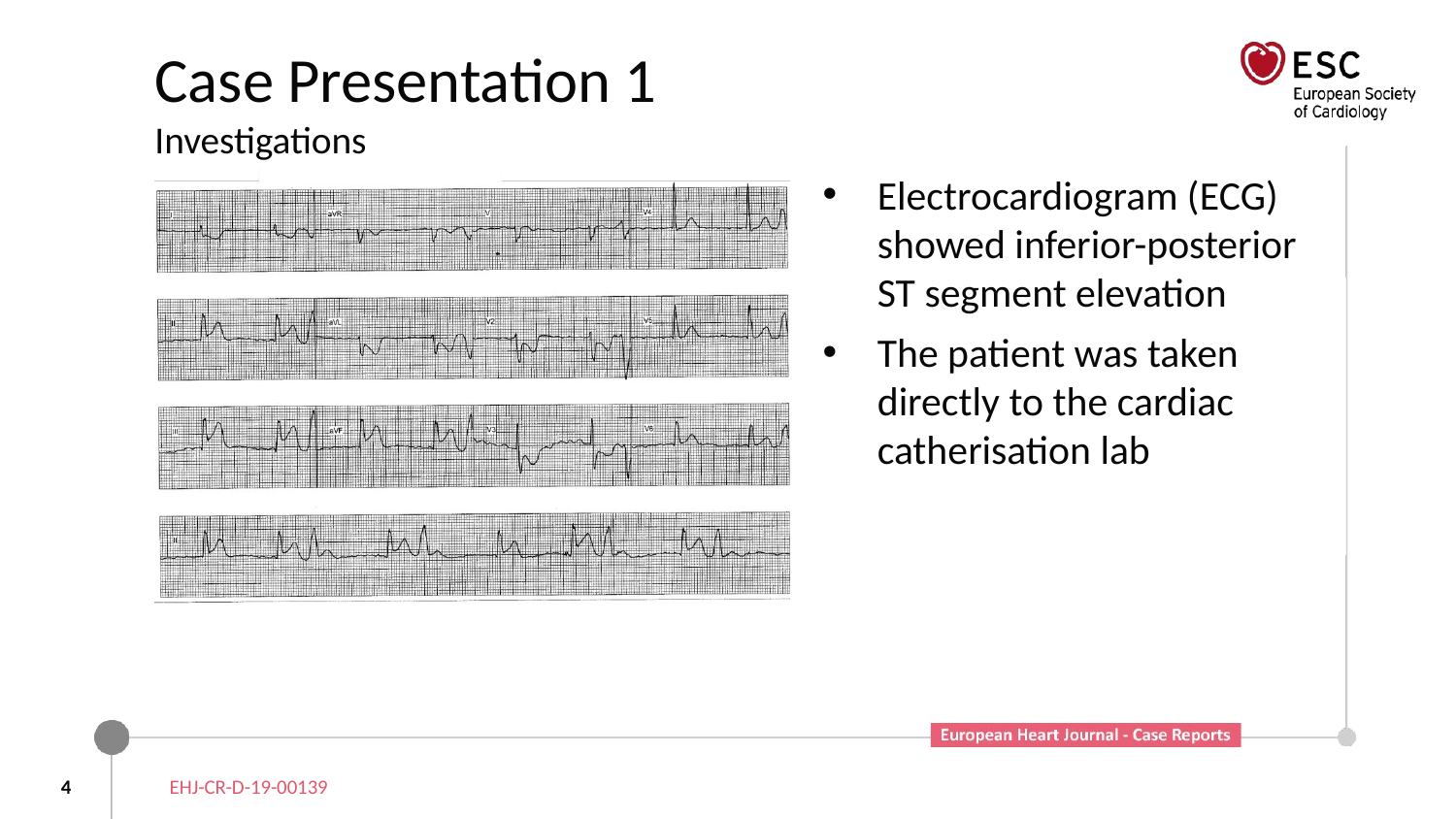

# Case Presentation 1 Investigations
Electrocardiogram (ECG) showed inferior-posterior ST segment elevation
The patient was taken directly to the cardiac catherisation lab
4
EHJ-CR-D-19-00139

## Slide 5
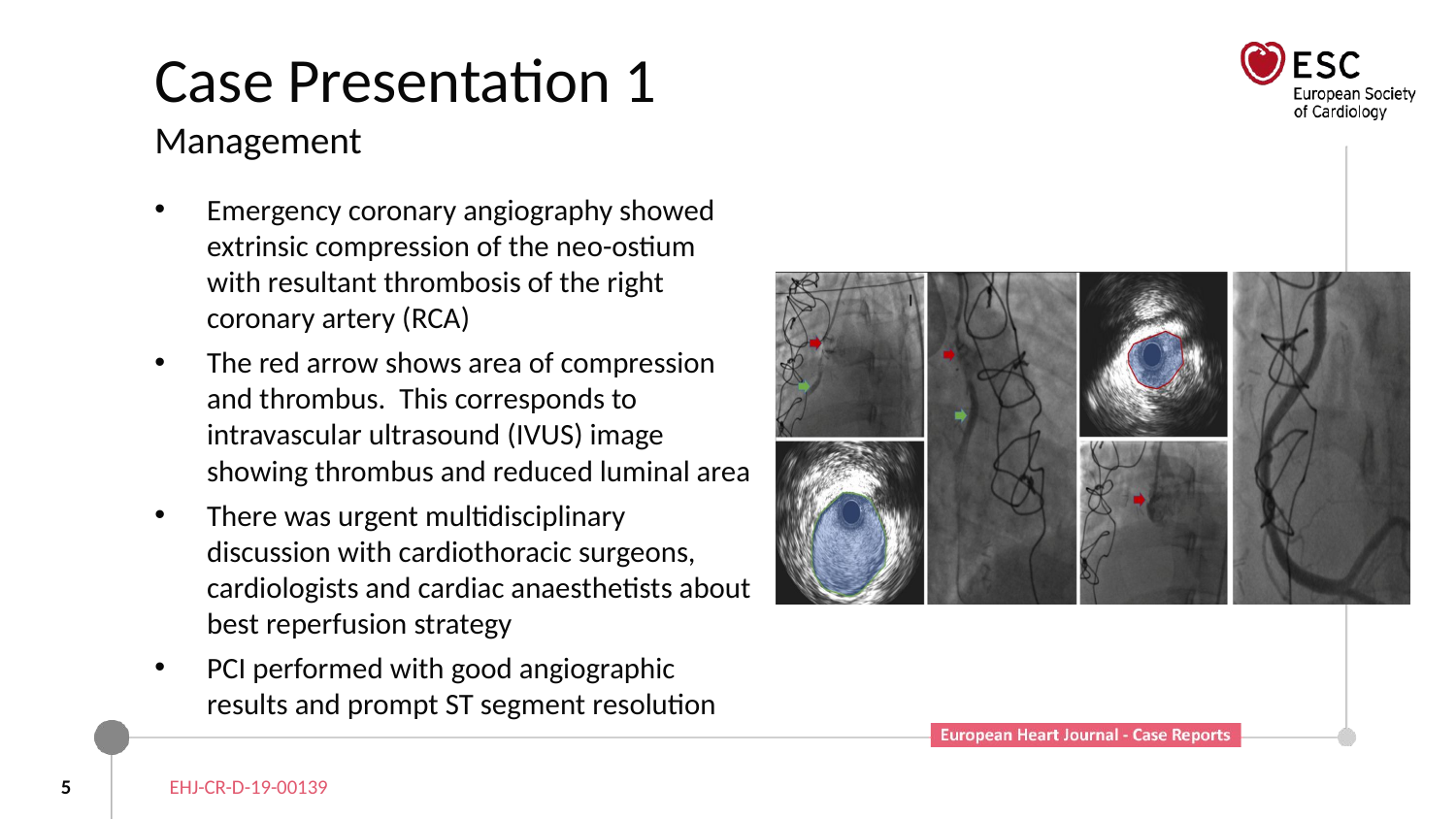

# Case Presentation 1 Management
Emergency coronary angiography showed extrinsic compression of the neo-ostium with resultant thrombosis of the right coronary artery (RCA)
The red arrow shows area of compression and thrombus. This corresponds to intravascular ultrasound (IVUS) image showing thrombus and reduced luminal area
There was urgent multidisciplinary discussion with cardiothoracic surgeons, cardiologists and cardiac anaesthetists about best reperfusion strategy
PCI performed with good angiographic results and prompt ST segment resolution
5
EHJ-CR-D-19-00139

## Slide 6
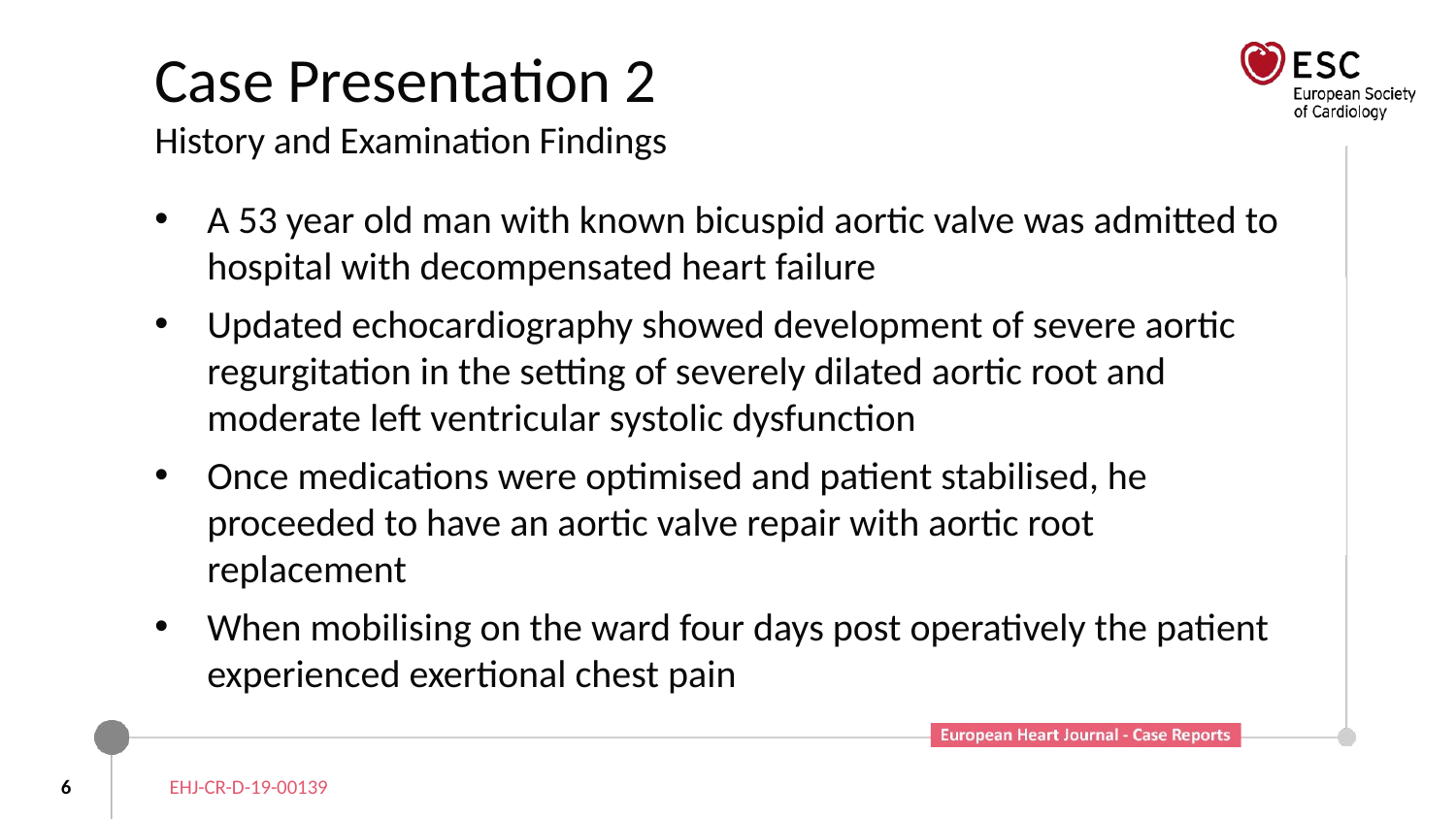

# Case Presentation 2 History and Examination Findings
A 53 year old man with known bicuspid aortic valve was admitted to hospital with decompensated heart failure
Updated echocardiography showed development of severe aortic regurgitation in the setting of severely dilated aortic root and moderate left ventricular systolic dysfunction
Once medications were optimised and patient stabilised, he proceeded to have an aortic valve repair with aortic root replacement
When mobilising on the ward four days post operatively the patient experienced exertional chest pain
6
EHJ-CR-D-19-00139

## Slide 7
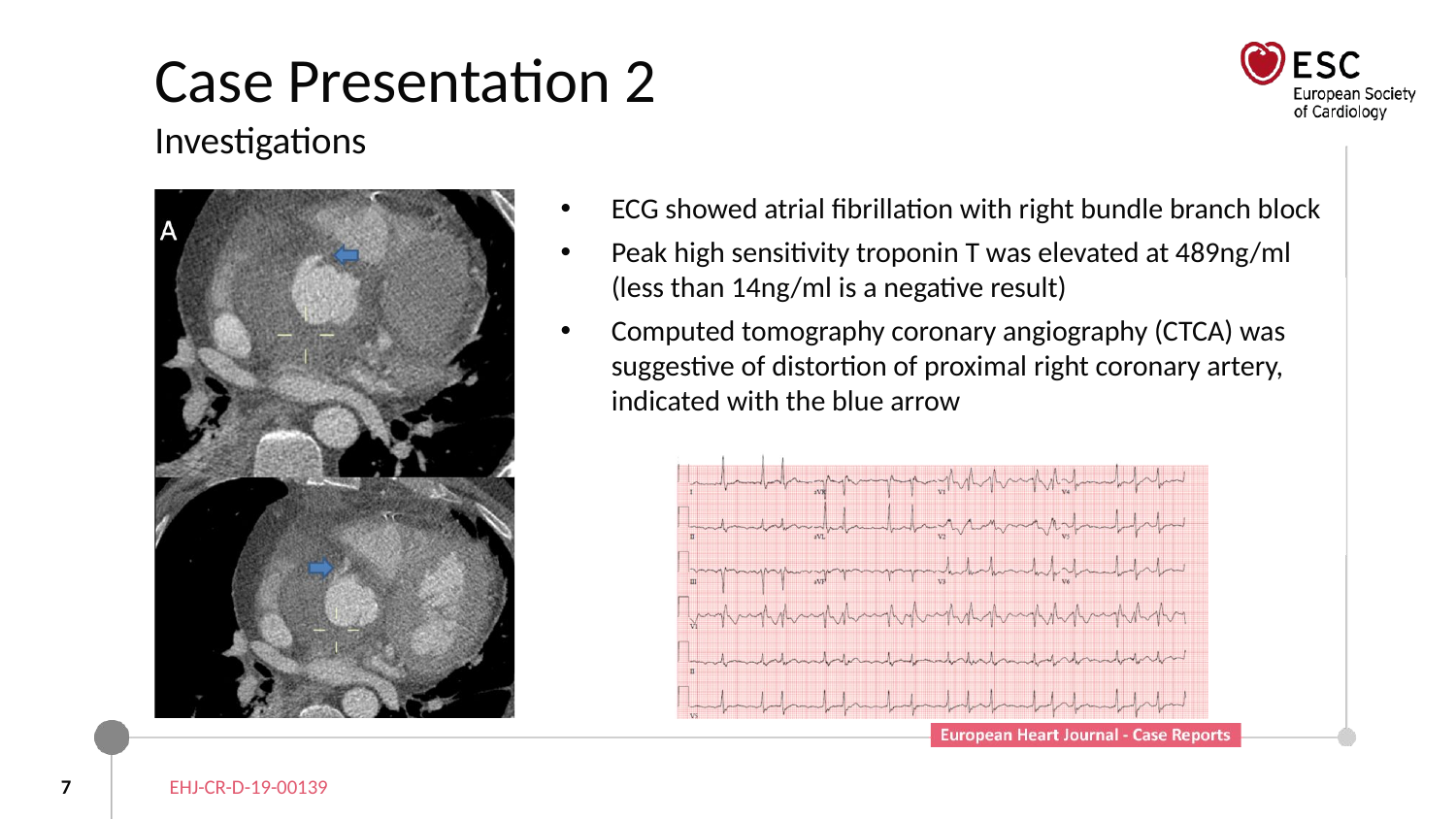

# Case Presentation 2 Investigations
ECG showed atrial fibrillation with right bundle branch block
Peak high sensitivity troponin T was elevated at 489ng/ml (less than 14ng/ml is a negative result)
Computed tomography coronary angiography (CTCA) was suggestive of distortion of proximal right coronary artery, indicated with the blue arrow
7
EHJ-CR-D-19-00139

## Slide 8
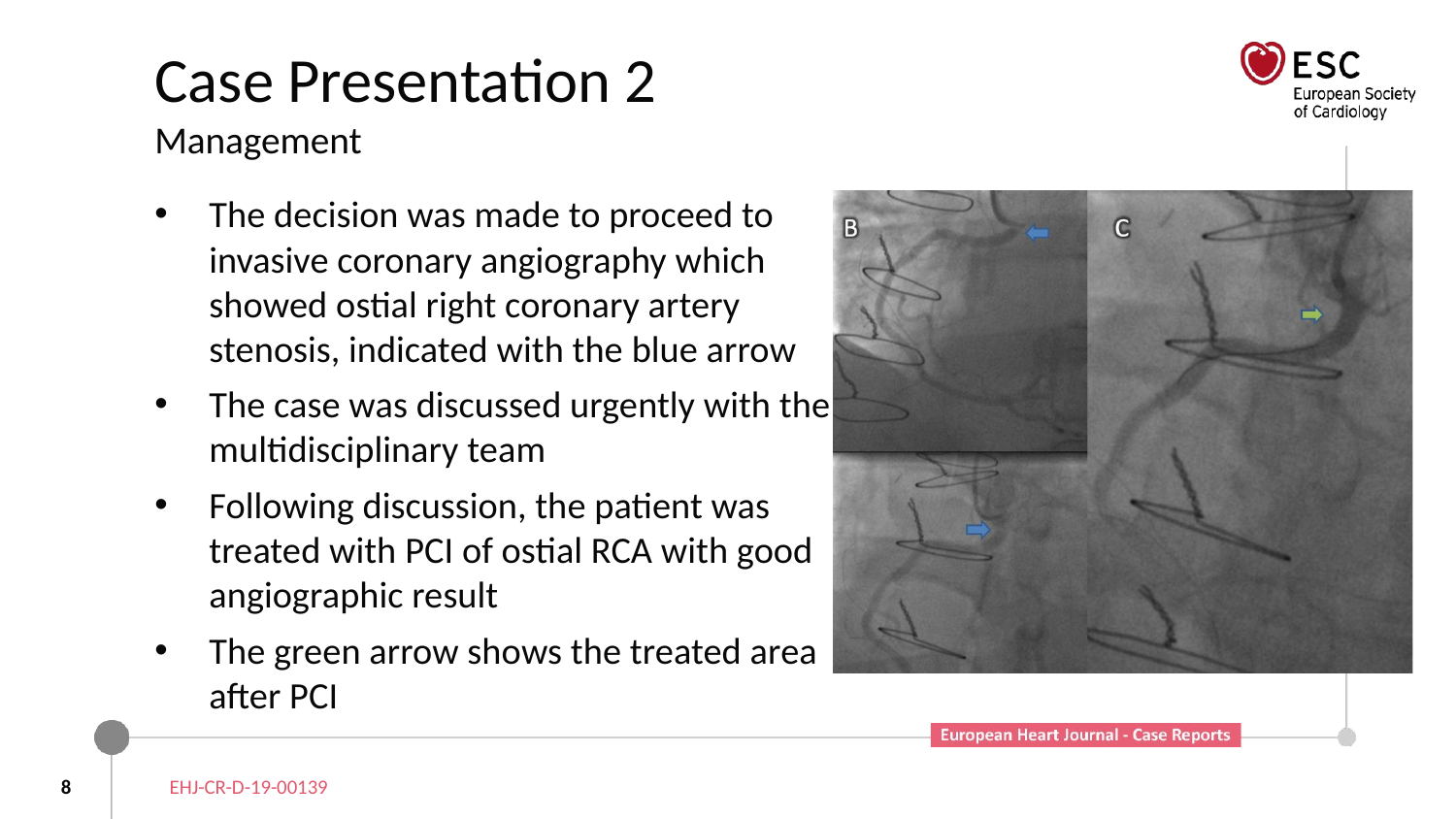

# Case Presentation 2 Management
The decision was made to proceed to invasive coronary angiography which showed ostial right coronary artery stenosis, indicated with the blue arrow
The case was discussed urgently with the multidisciplinary team
Following discussion, the patient was treated with PCI of ostial RCA with good angiographic result
The green arrow shows the treated area after PCI
8
EHJ-CR-D-19-00139

## Slide 9
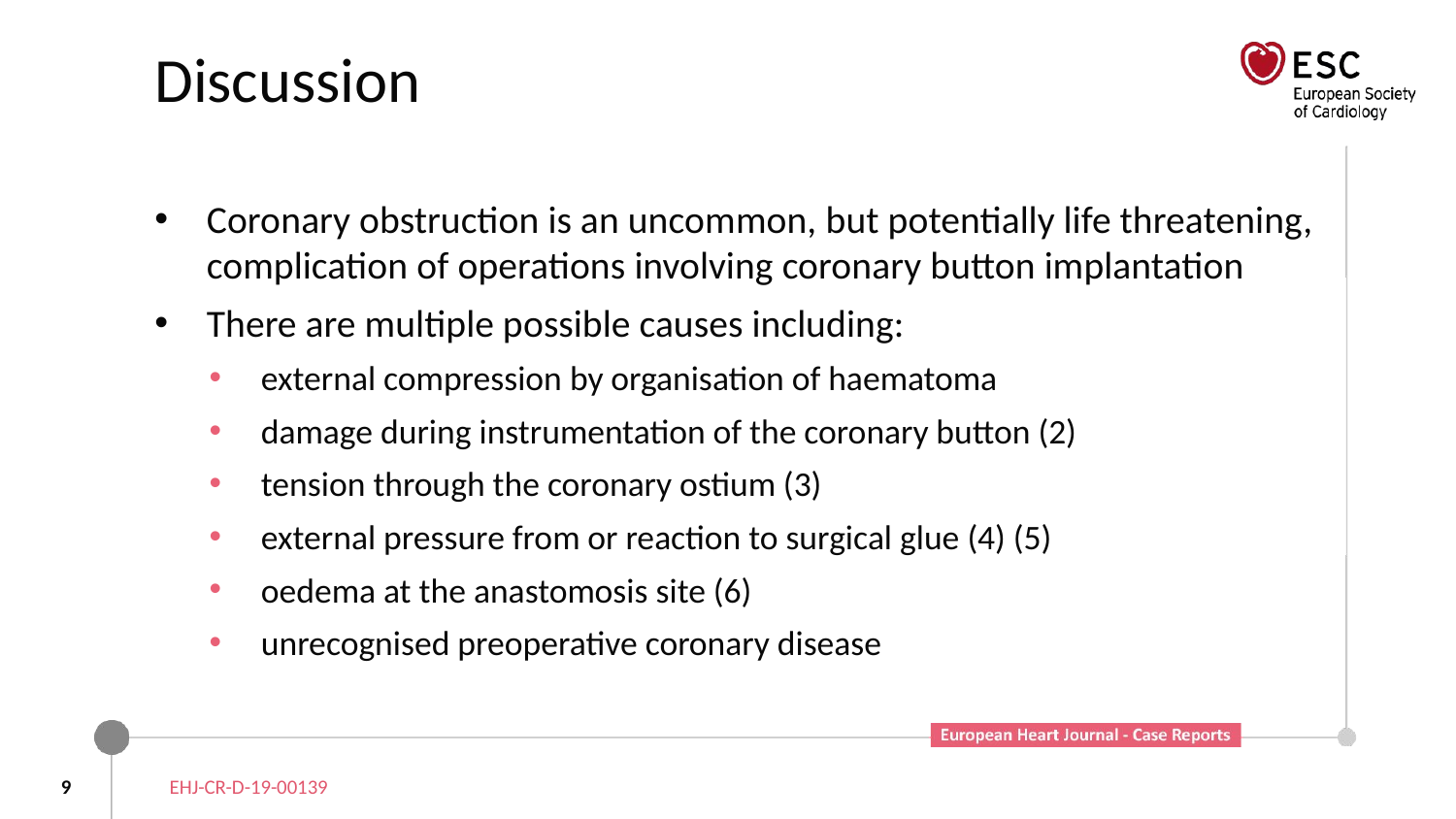

# Discussion
Coronary obstruction is an uncommon, but potentially life threatening, complication of operations involving coronary button implantation
There are multiple possible causes including:
external compression by organisation of haematoma
damage during instrumentation of the coronary button (2)
tension through the coronary ostium (3)
external pressure from or reaction to surgical glue (4) (5)
oedema at the anastomosis site (6)
unrecognised preoperative coronary disease
9
EHJ-CR-D-19-00139

## Slide 10
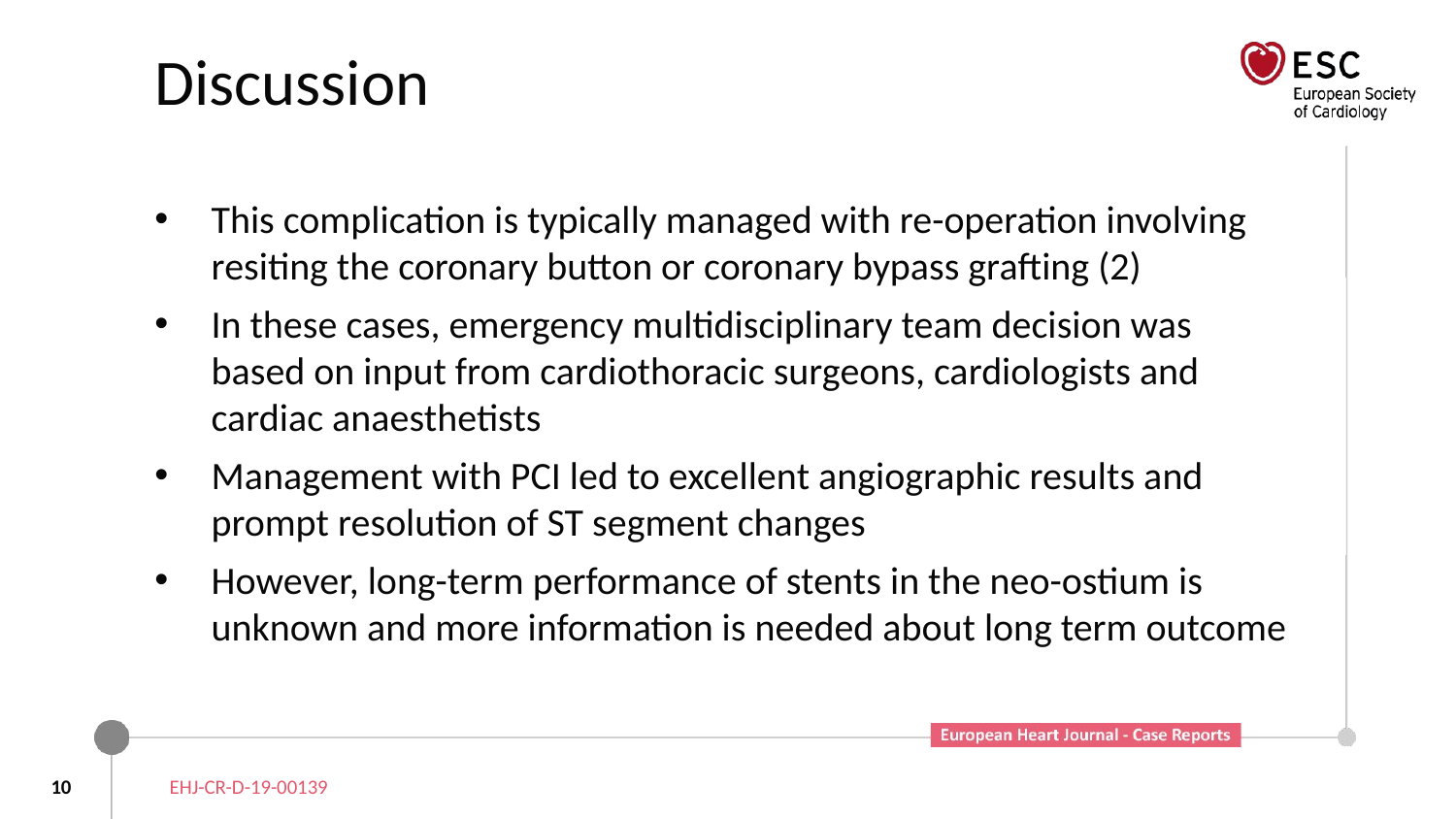

# Discussion
This complication is typically managed with re-operation involving resiting the coronary button or coronary bypass grafting (2)
In these cases, emergency multidisciplinary team decision was based on input from cardiothoracic surgeons, cardiologists and cardiac anaesthetists
Management with PCI led to excellent angiographic results and prompt resolution of ST segment changes
However, long-term performance of stents in the neo-ostium is unknown and more information is needed about long term outcome
10
EHJ-CR-D-19-00139

## Slide 11
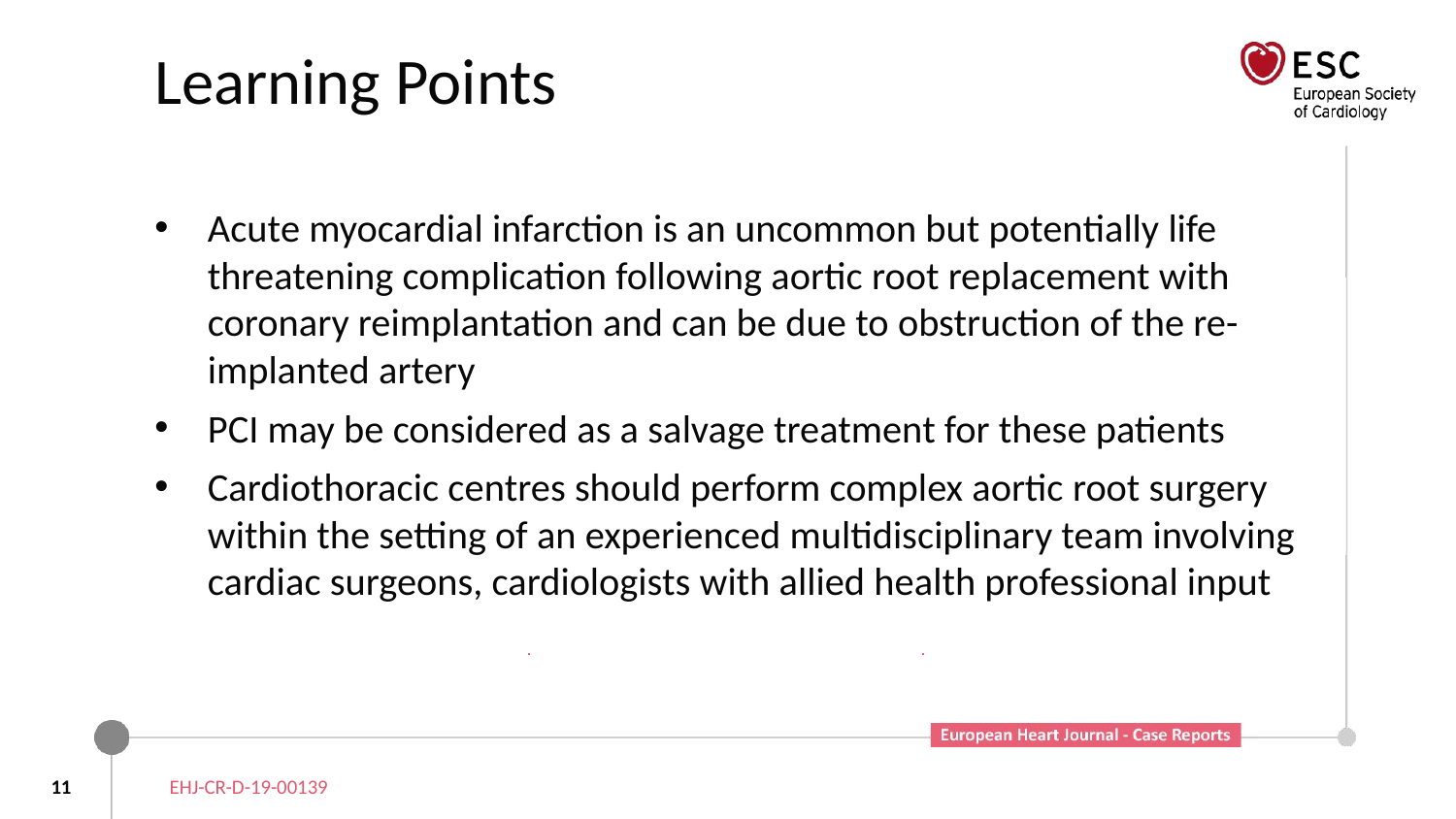

# Learning Points
Acute myocardial infarction is an uncommon but potentially life threatening complication following aortic root replacement with coronary reimplantation and can be due to obstruction of the re-implanted artery
PCI may be considered as a salvage treatment for these patients
Cardiothoracic centres should perform complex aortic root surgery within the setting of an experienced multidisciplinary team involving cardiac surgeons, cardiologists with allied health professional input
11
EHJ-CR-D-19-00139

## Slide 12
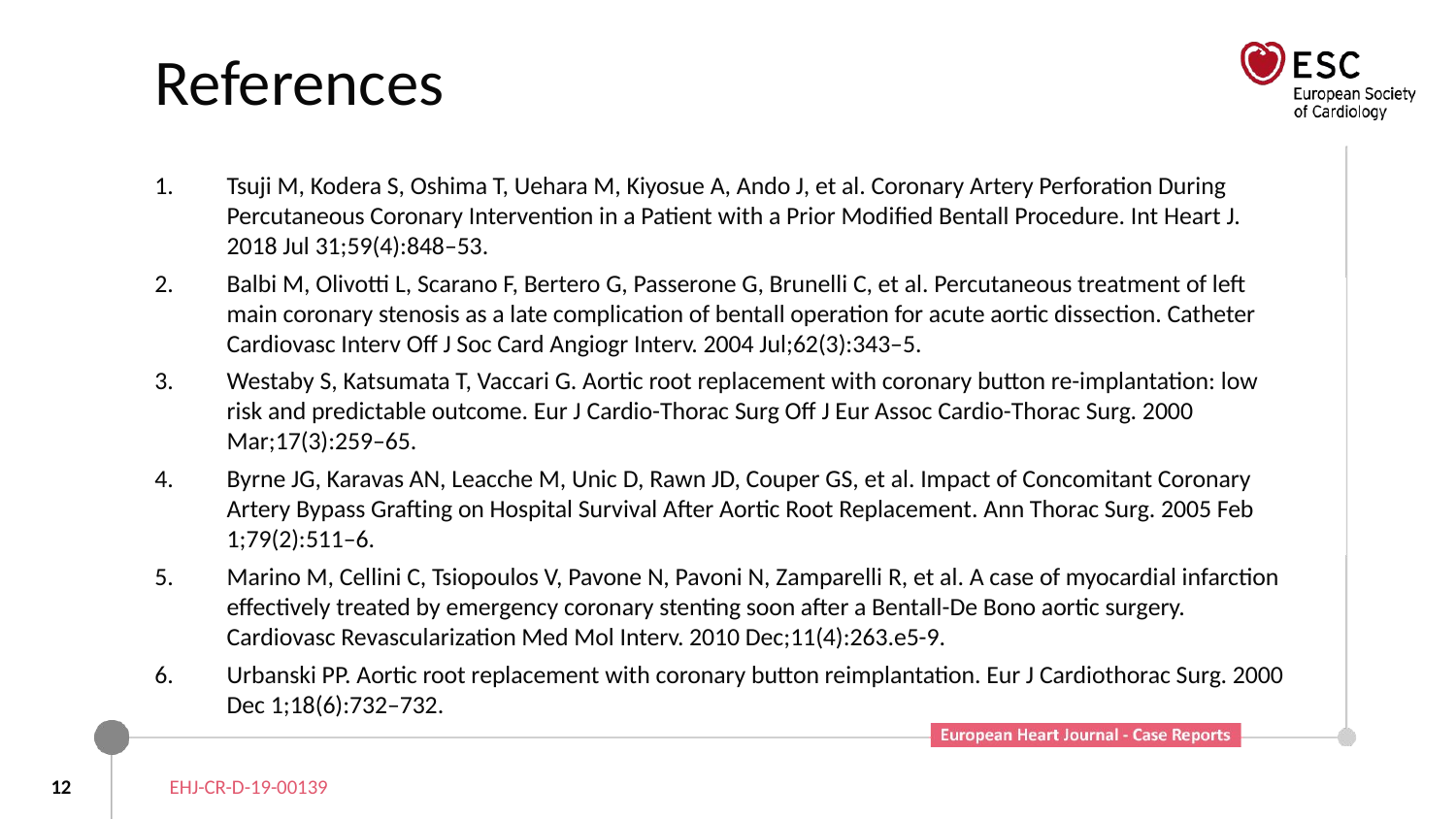

# References
Tsuji M, Kodera S, Oshima T, Uehara M, Kiyosue A, Ando J, et al. Coronary Artery Perforation During Percutaneous Coronary Intervention in a Patient with a Prior Modified Bentall Procedure. Int Heart J. 2018 Jul 31;59(4):848–53.
Balbi M, Olivotti L, Scarano F, Bertero G, Passerone G, Brunelli C, et al. Percutaneous treatment of left main coronary stenosis as a late complication of bentall operation for acute aortic dissection. Catheter Cardiovasc Interv Off J Soc Card Angiogr Interv. 2004 Jul;62(3):343–5.
Westaby S, Katsumata T, Vaccari G. Aortic root replacement with coronary button re-implantation: low risk and predictable outcome. Eur J Cardio-Thorac Surg Off J Eur Assoc Cardio-Thorac Surg. 2000 Mar;17(3):259–65.
Byrne JG, Karavas AN, Leacche M, Unic D, Rawn JD, Couper GS, et al. Impact of Concomitant Coronary Artery Bypass Grafting on Hospital Survival After Aortic Root Replacement. Ann Thorac Surg. 2005 Feb 1;79(2):511–6.
Marino M, Cellini C, Tsiopoulos V, Pavone N, Pavoni N, Zamparelli R, et al. A case of myocardial infarction effectively treated by emergency coronary stenting soon after a Bentall-De Bono aortic surgery. Cardiovasc Revascularization Med Mol Interv. 2010 Dec;11(4):263.e5-9.
Urbanski PP. Aortic root replacement with coronary button reimplantation. Eur J Cardiothorac Surg. 2000 Dec 1;18(6):732–732.
12
EHJ-CR-D-19-00139
